# Fluid Motion and Frozen Time

**DOI:** 10.3201/eid2706.AC2706

**Published:** 2021-06

**Authors:** Byron Breedlove

**Affiliations:** Centers for Disease Control and Prevention, Atlanta, Georgia, USA

**Keywords:** art science connection, emerging infectious diseases, art and medicine, about the cover, Thomas Red Owl Haukaas, More Time Expected, Fluid Motion and Frozen Time, American art, Pneumocystis pneumonia, HIV/AIDS and other retroviruses, viruses, sexually transmitted infections, pandemic, Native Americans

**Figure:**
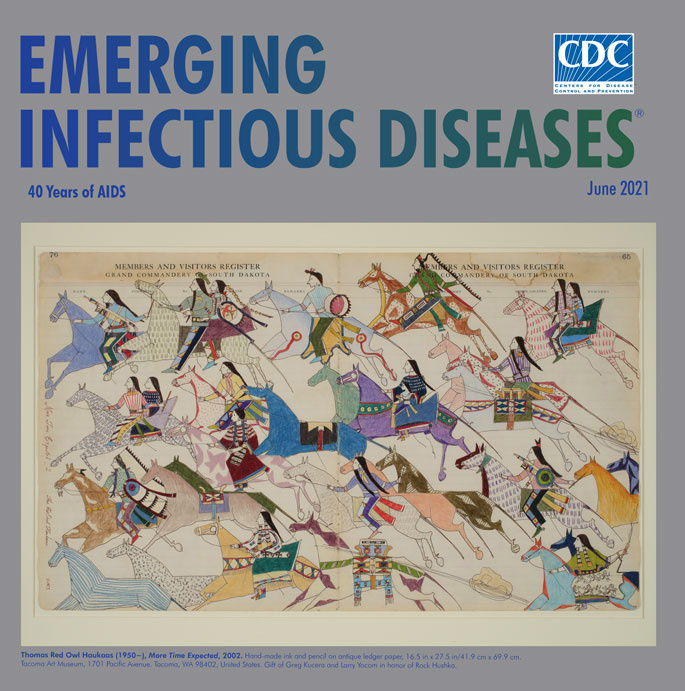
**Thomas Red Owl Haukaas (1950**−), ***More Time Expected*, 2002.** Hand-made ink and pencil on antique ledger paper, 16.5 in × 27.5 in/41.9 cm × 69.9 cm. Tacoma Art Museum, 1701 Pacific Avenue, Tacoma, WA 98402, United States. Gift of Greg Kucera and Larry Yocoreem in honor of Rock Hushka.

In June 1981, five cases of *Pneumocystis* pneumonia in gay men were described in CDC’s *Morbidity and Mortality Weekly Report*. Those cases signaled the start of the AIDS pandemic, which now enters its fifth decade and has to date resulted in more than 75 million HIV infections and 32 million deaths worldwide. UNAIDS estimates that in 2019, 38 million persons were living with HIV, 1.7 million became newly infected, and 690,000 died with HIV disease.

Since the beginning of the HIV/AIDS pandemic, artists―some involved in AIDS activist organizations and others working independently―have applied their talents and skills to create, share, and deliver works that depict messages calling for political action and scientific research, documenting the impact of AIDS among various and diverse communities and groups, and celebrating medical breakthroughs and advances in treating AIDS. In 2016, a traveling exhibition entitled *Art AIDS America* examined the ongoing influence and impact of the AIDS pandemic on American art. 

Among the more than 125 works featured in that traveling exhibition was *More Time Expected* by Lakota artist Thomas Red Owl Haukaas, displayed on this month’s cover. Haukaas conveys both a sense of fluid motion and frozen time in this image, a modern example of Native American ledger art (a genre of narrative drawing or painting on paper or cloth) that developed during the Indian Wars era and continued in the forced relocations of Plains tribes to government reservations from the 1860s through the 1920s. Widespread hunting had depleted buffalo and other game animals that provided hides the tribes traditionally used as canvases for recording events, ceremonies, and exploits. As a result, Native Americans began using paper taken from ledgers and other sources and employing ink, pencils, and watercolors rather than bone or wooden implements dipped in mineral and other natural pigments. 

In this work, Haukaas shows a group of Native Americans and horses sweeping from right to left across the paper. The figures and horses are crowded, flattened, and overlapping each other. Riders and horses are looking straight ahead and moving in unison toward a destination beyond the edge of the image. The artist carefully depicts his figures of Native Americans dressed in traditional garb and wearing an array of bright colors and patterns. The horses are also stylized and individualized: many are boldly colored, and others are sketched with repeating patterns and rows of stripes. Half of the horses carry either a single rider or a pair of riders, but the other half are riderless, including the cobalt blue horse that draws attention to the center of the image. 

Michelle Reynolds, Associate Director of Marketing and Communications at the Tacoma Art Museum, which organized *Art AIDS America* in partnership with the Bronx Museum of the Arts, explains how this work is related to HIV/AIDS: “The imagery, specifically the riderless horse, explores the complicated issues of stigma surrounding HIV/AIDS and the Native American experience with the disease. Historically, instances of HIV on the reservation are virtually unmentioned, a silence that only worsens an already high rate of infection. The horse with no rider, often used as a symbol for a warrior who fell in battle, represents individuals on the reservation who have died of AIDS-related causes. By focusing on absence within a group, Haukaas plays to the importance of community and families within these settings.”

In a 2018 interview, Haukaas told writer and editor Emily Withnall, “My pieces are meant for dialogue, for discussion, for thinking about.” Despite the fluidity and motion of his work, the underlying message carried by his portrayal of these riderless horses is the palpable sense of what has been lost. Haukaas, whose works are featured in many museum collections and have been part of numerous exhibitions, does not have formal training in art, and he credits his family and friends for teaching him traditional skills and practices. Known for his talents as a ledger, beadwork, and doll artist, he trained as a psychiatrist. 

Through this image, Haukaas reconnects with the traditional ledger art form and uses it as a platform to engender thought and discussion about the ongoing medical and social effects of HIV within reservation communities, throughout other marginalized racial and ethnic communities around the world, and in those regions most affected by this ongoing pandemic. As De Cock, Jaffe, and Curran state in their EID article Reflections on 40 Years of AIDS, “Although initially slow, the HIV/AIDS response over the years has been a beacon in global health for respect for individuals and their rights and for health equity. More reflection is required with regard to what the responses to HIV and Ebola have taught us and how they might be relevant to COVID-19 and other future epidemics.”
